# Comparison of the QuantiGene 2.0 Assay and Real-Time RT-PCR in the Detection of p53 Isoform mRNA Expression in Formalin-Fixed Paraffin-Embedded Tissues- A Preliminary Study

**DOI:** 10.1371/journal.pone.0165930

**Published:** 2016-11-10

**Authors:** Brianna C. Morten, Rodney J. Scott, Kelly A. Avery-Kiejda

**Affiliations:** 1 Medical Genetics, Hunter Medical Research Institute, New Lambton Heights, NSW, 2305, Australia; 2 Priority Research Centre for Cancer, School of Biomedical Sciences and Pharmacy, Faculty of Health and Medicine, University of Newcastle, Newcastle, NSW, 2308, Australia; 3 Pathology North, John Hunter Hospital, New Lambton Heights, NSW, 2305, Australia; Bauer Research foundation, UNITED STATES

## Abstract

p53 is expressed as multiple smaller isoforms whose functions in cancer are not well understood. The p53 isoforms demonstrate abnormal expression in different cancers, suggesting they are important in modulating the function of full-length p53 (FLp53). The quantification of relative mRNA expression has routinely been performed using real-time PCR (qPCR). However, there are serious limitations when detecting p53 isoforms using this method, particularly for formalin-fixed paraffin-embedded (FFPE) tissues. The use of FFPE tumours would be advantageous to correlate expression of p53 isoforms with important clinical features of cancer. One alternative method of RNA detection is the hybridization-based QuantiGene 2.0 Assay, which has been shown to be advantageous for the detection of RNA from FFPE tissues. In this pilot study, we compared the QuantiGene 2.0 Assay to qPCR for the detection of FLp53 and its isoform Δ40p53 in matched fresh frozen (FF) and FFPE breast tumours. FLp53 mRNA expression was detected using qPCR in FF and FFPE tissues, but Δ40p53 mRNA was only detectable in FF tissues. Similar results were obtained for the QuantiGene 2.0 Assay. FLp53 relative mRNA expression was shown to be strongly correlated between the two methods (R^2^ = 0.9927, p = 0.0031) in FF tissues, however Δ40p53 was not (R^2^ = 0.4429, p = 0.3345). When comparing the different methods for the detection of FLp53 mRNA from FFPE and FF samples, no correlation (R^2^ = 0.0002, p = 0.9863) was shown using the QuantiGene 2.0 Assay, and in contrast, the level of expression was highly correlated between the two tissues using qPCR (R^2^ = 0.8753, p = 0.0644). These results suggest that both the QuantiGene 2.0 Assay and qPCR methods are inadequate for the quantification of Δ40p53 mRNA in FFPE tissues. Therefore, alternative methods of RNA detection and quantification are required to study the relative expression of Δ40p53 in FFPE samples.

## Introduction

Formalin-fixed paraffin embedded (FFPE) tissue samples have been collected for thousands of breast cancer specimens with substantial clinical information for diagnostic investigation. RNA isolation from these specimens has allowed clinical information to be correlated with gene expression to identify novel biomarkers of disease [1, 2]. However, the quality of the mRNA can be heavily degraded in FFPE tissues, and as a result there are serious limitations in studying the relative mRNA expression in these samples [3–5].

Advances in mRNA quantitation are providing novel opportunities for quantitative gene expression analysis in FFPE tissues. One such method is the QuantiGene 2.0 Assay by Panomics [6]. This hybridization based assay utilises branched DNA capture Probe Sets that conjugate to target mRNA to amplify a signal, rather than amplification of a direct target. The probe-based assay uses Z-probe structures which binds to smaller 20bp regions consecutively to generate a larger targeted region, compared to a primer-probe amplicon used in real-time PCR. This assay is therefore advantageous for the detection of highly degraded RNA from FFPE tissues, as the manner in which the probes consecutively bind to the sequence allows for high specificity, and it does not require any reverse transcription or mRNA processing and therefore avoids any loss of signal, or inaccuracies, that may result from chemical modification or degraded material caused by tissue fixation [4].

Small transcript variants of p53 have been reported, with 14 different p53 isoforms identified to date [7–9]. We recently reported that Δ40p53 is the most highly expressed p53 isoform in a cohort of 148 fresh frozen (FF) breast tumour tissues [10]. Further examination of the relative mRNA expression of the p53 isoforms, including Δ40p53, may be important in relation to clinical features of breast cancer, treatment responses and prognosis.

Previously, we have designed custom Taqman^®^ probes to quantitate the mRNA expression levels of the p53 isoforms from FF breast tumour samples using traditional real-time PCR methods [10]. However, due to the limitations in real-time PCR primer and probe design, the amplicon size was too large to allow for investigation of the p53 isoforms in FFPE tissues. In this study, we custom designed probe sets through Panomics to measure the Δ40p53 and full-length p53 (FLp53) isoforms using the QuantiGene 2.0 Assay. We tested this assay on a pilot group of four breast tumour samples to quantify p53 isoform mRNA expression from FFPE tissues, and compared this to the mRNA expression in matched FF samples analysed by real-time PCR. This method would ideally be used to quantify low level mRNA expression, as observed with p53 isoforms from FFPE tumour samples, and would overcome the restrictions on amplicon length that we faced using real-time RT-PCR. Our results showed that the QuantiGene 2.0 Assay was not comparable to real-time PCR for the detection of FLp53 mRNA from FFPE samples, compared to matched FF tissue, and that Δ40p53 mRNA expression could not be quantitated using either method. This suggests that neither method is appropriate for quantitating the relative mRNA expression of Δ40p53 from FFPE tissues.

## Materials and Methods

### Study cohort

Four matched breast tumour samples were provided by the Australian Breast Cancer Tissue Bank (Westmead, Sydney, NSW, Australia), from either FF or FFPE collections. All tumour samples have been described previously [10]. This study complies with the Helsinki Declaration with ethical approval from the Hunter New England Human Research Ethics Committee (Approval number: 09/05/20/5.02). All patients have given written consent for their tissue specimens and clinical information to be stored at the Australian Breast Cancer Tissue Bank for distribution and use in ethically approved research projects. RNA was extracted from the FF samples using the RNeasy kit (Qiagen, VIC, Australia). RNA was also extracted from a 2mm core, from a tumour-rich area as identified by a pathologist, using the RNeasy FFPE- kit (Qiagen). RNA was quantified using the Quant-it RiboGreen RNA Assay kit (Life Technologies, Mulgrave, VIC, Australia) and purity assessed by A_260/A280_ ratios (>1.8) using the Nanodrop (Thermo Scientific, Wilmington, DE, USA). The RNA integrity of tumour samples was analysed using the 2100 Bioanalyser and the RNA 6000 Nano kit (Agilent Technologies, Mulgrave, VIC, Australia) and all fresh-frozen samples used in this analysis had an RNA Integrity Number (RIN) of 8 or greater.

### Semi-quantitative real-time PCR

Total RNA (660ng) was reverse transcribed to generate cDNA using the High Capacity cDNA Reverse Transcription Kit (Life Technologies, Mulgrave, VIC, Australia) according to the manufacturers’ instructions. Real-time PCR analysis was performed in triplicate using TaqMan^®^ Universal PCR mix (Life Technologies) on 20ng (tumour samples) according to the manufacturers’ instructions, with results quantified on a 7500 real-time PCR system (Life Technologies). The expression of Δ40p53 and FLp53 was analysed as previously described [10]. Taqman Gene Expression Assays for β-Actin (Human ACTB Endogenous Control) and β2-microglobulin (Assay ID: Hs99999907_m1) were also used (Life Technologies). PCR reactions containing no cDNA and no reverse transcriptase were included in every PCR run. The relative expression of Δ40p53 and FLp53 was normalised to β-Actin or β2-microglobulin (ΔCt) and expressed as the fold change calculated using the 2^-ΔΔCt^ method as described previously [11]. The relative expression of β-Actin in FF tumours was not significantly different between the tumours analysed and therefore served as an appropriate normaliser for this analysis.

### QuantiGene 2.0 Assay

Total RNA was quantified using custom-designed Probe Sets for three genes; Δ40p53, FLp53 and the housekeeping gene, β-Actin using the QuantiGene 2.0 Assay (Panomics, Affymetrix, Santa Clara, CA, USA) in triplicate. For more information about the Probe Sets, see Table 1. Briefly, 100ng of total RNA was dispensed into capture plates in triplicate for each probe set used (Δ40p53, FLp53 and β-Actin). Working probe sets were added for each target gene, which included capture extenders, blocking probes and label extenders, and were hybridised to the target RNA overnight at 55°C. The probed target mRNA was amplified via a series of hybridizations of pre-amplifier, amplifier and label probe structures to the bound probe sets. Signal amplification of the target RNA was measured by the addition of a chemiluminescent substrate, which produced a luminescent signal based on the number of hybridised label probes per well. The level of luminescence reported was proportional to the number of target mRNA molecules present in the sample. Luminescence was measured using the FLUOstar Optima plate reader (BMG Labtech, Mornington, VIC, Australia). An assay background control was also performed in triplicate for each probe set analysed (S1 Fig). Data was interpreted as normalised gene expression by dividing the background-subtracted average signals by the background-subtracted average signal of the housekeeping gene.

**Table 1:** Location of the Probe Sets designed for the target genes used in the QuantiGene 2.0 Assay.

Gene	Region covered by Probe Set (bp)	Accession number
Δ40p53	106–472	NM_001126118
FLp53	128–511	NM_000546
Β-Actin	296–822	NM_001101

### Statistical analysis

Linear regression analysis was performed to determine the correlative relationship of the relative mRNA expression of the different genes between FFPE and FF samples, using the two different methods described above. Firstly, a linear regression analysis was performed to compare the relative expression of normalised Δ40p53 and FLp53 expression from FF-derived RNA using real-time PCR and the QuantiGene 2.0 Assay. Following this, the relative expression from the FF samples was compared to that from the FFPE samples using the QuantiGene 2.0 Assay and real-time PCR. A Pearson’s correlation coefficient test was used to calculate the significance (p <0.05) between the different assays and samples. All results are presented as the mean ± SEM, with each bar representing three independent experiments. All statistical analyses were performed using GraphPad Prism v6.0 software (GraphPad Software, La Jolla, CA, USA).

## Results

In order to determine the most appropriate method for the analysis of Δ40p53 and FLp53 mRNA expression from both FF and FFPE tumour tissue samples, the two different quantification methods of real-time PCR and the QuantiGene 2.0 Assay were compared. The two methods were used to compare the expression of Δ40p53 and FLp53 in four matched breast cancer samples from either FFPE or FF tissue.

### Signal detection using either the QuantiGene 2.0 Assay or real-time PCR

**Fig 1:**
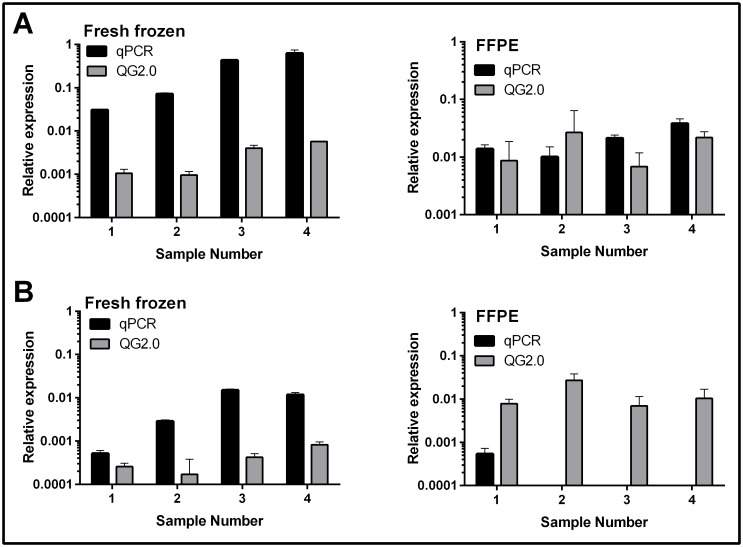
Comparison of the relative mRNA expression of Δ40p53 and FLp53 using the QuantiGene 2.0 Assay (QG 2.0) and real-time PCR (qPCR). (A) The relative expression of (A) FLp53 or (B) Δ40p53 from either FF or FFPE tissues, using real-time PCR (qPCR; black) or the QuantiGene 2.0 Assay (QG2.0; grey). Sample quantitation using the QuantiGene 2.0 Assay was performed in triplicate and normalised to β-Actin, and FFPE samples using real-time PCR were normalised to β2-Microglobulin.The bars represent the mean ± SD.

The signal detected for FLp53 mRNA was shown to be comparable between both methods tested in the FF samples (Fig 1A, Table 2). The QuantiGene 2.0 Assay measures gene expression by generating a luminescent signal which is proportional to the amount of target mRNA present in any given sample. The plate can be read using a multi-mode plate reader which records the relative luminescent unit (RLU) per well (S1 Fig). FLp53 was also detected in the FFPE samples using both assays (Fig 1A, Table 2, S2 and S4 Figs). However, there were clear differences in the detected signal for Δ40p53 mRNA, where Δ40p53 was detected in the FF samples using both methods, but was unable to be detected using real-time PCR in the FFPE samples (Fig 1B, Table 2, S2 and S3 Figs). This was expected given the amplicon size of Δ40p53 in the real-time PCR assay (147bp) was too large to allow for the investigation of this p53 isoform in FFPE tissues.

**Table 2:** Average detected signal for three genes tested in fresh frozen (FF) and FFPE tissues.

Tumour input	Sample number	Average signal using QG 2.0 (%CV)			Average expression (2^-Ct^) using real-time PCR (%CV)		
		**β-Actin**	**Δ40p53**	**FLp53**	**β-Actin**	**Δ40p53**	**FLp53**
**Fresh Frozen**	1	192972 (1.64)	183 (5.32)	417 (11.29)	1.41E-05 (3.89)	7.24E-09 (16.60)	4.3E-07 (5.99)
	2	234610 (4.15)	156 (29.32)	439 (10.60)	6.50E-06 (10.21)	1.87E-08 (6.97)	4.64E-07 (6.58)
	3	143540 (6.98)	194 (6.74)	788 (11.89)	4.11E-06 (38.24)	5.79E-08 (6.82)	1.67E-06 (3.17)
	4	115378 (0.35)	228 (6.85)	873 (1.89)	3.09E-06 (7.18)	3.60E-08 (12.46)	1.93E-06 (19.71)
		**β-Actin**	**Δ40p53**	**FLp53**	**β2-Microglobulin**	**Δ40p53**	**FLp53**
**FFPE**	1	8083 (1.67)	196 (8.43)	256 (28.50)	1.13E-07 (19.69)	5.99E-11 (35.09)	1.54E-09 (17.61)
	2	2626 (4.36)	202 (13.73)	253 (32.37)	6.47E-09 (11.08)	Undetermined	6.52E-11 (49.06)
	3	4864 (7.08)	166 (12.93)	246 (9.64)	8.08E-08 (11.06)	Undetermined	1.72E-09 (12.70)
	4	4858 (3.76)	162 (25.98)	317 (8.83)	5.47E-08 (14.67)	Undetermined	2.08E-09 (20.58)
**Limit of Detection**		126.58	255.50	262.26			

The average detection signal, percentage of the coefficient of variance (%CV) and limit of detection was measured for β-Actin, Δ40p53 and FLp53 using the QuantiGene 2.0 Assay. The average signal and the %CV was also measured for β2-microglobulin, Δ40p53 and FLp53 using real-time PCR, shown as the 2^-Ct^. Each sample was measured in triplicate for each gene.

Importantly, the average detected signal for Δ40p53 mRNA expression by the QuantiGene 2.0 Assay, in both the FF and FFPE-derived RNA, failed to reach the limit of detection for the assay (Table 2). This was also the case for FLp53 mRNA expression in three of the four FFPE samples analysed (Table 2.) There was also a lack of reproducibility between the technical replicates for the detection of FLp53 and Δ40p53 using the QuantiGene 2.0 Assay, as shown by the high coefficient of variance (%CV) in Table 2. The detected signal for the housekeeping gene, β-Actin, was well above the limit of detection in all samples but was significantly greater in the FF material compared to the FFPE using the QuantiGene 2.0 Assay, as would be expected given that FFPE-derived mRNA is heavily degraded (Table 2). This validates the use of the QuantiGene 2.0 Assay for genes that are highly expressed.

### Correlation between the QuantiGene 2.0 Assay and real-time PCR

**Fig 2:**
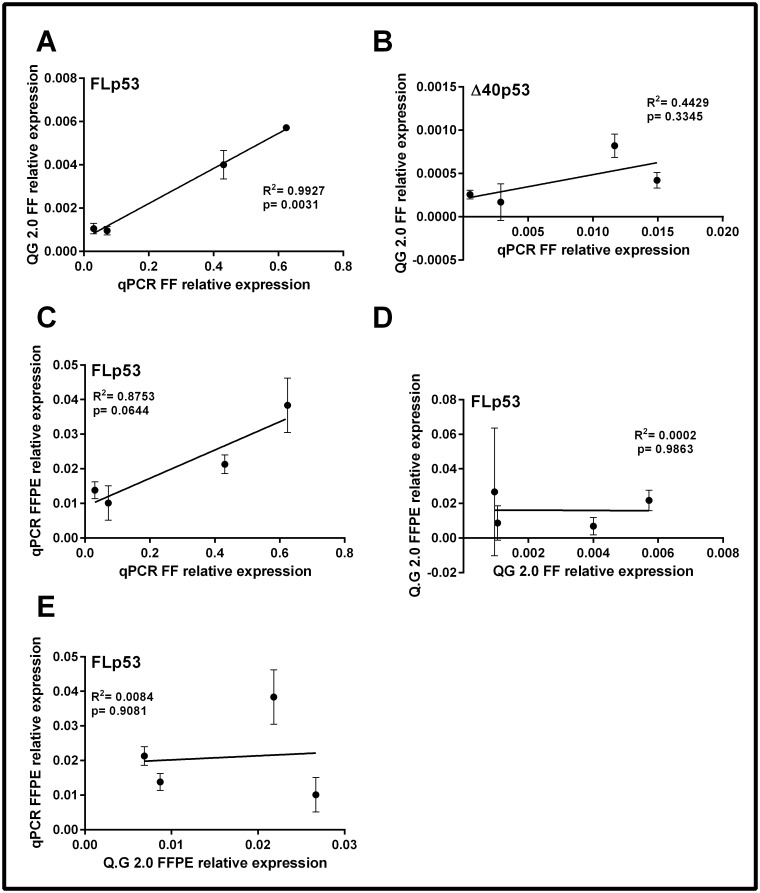
Correlation of FLp53 and Δ40p53 relative expression from FF and FFPE samples using two different quantification methods. A linear regression analysis was performed to compare the relative expression of FLp53 (A) and Δ40p53 (B) using real-time PCR (qPCR) and the QuantiGene 2.0 Assay (QG 2.0) with fresh frozen (FF) samples. The expression of FLp53 was also compared following qPCR (C) or QG 2.0 (D) between FF and FFPE samples, and between qPCR and QG 2.0 in FFPE samples only (E). All samples were quantitated in triplicate with data representing the mean ± SD.

In order to determine if the QuantiGene 2.0 Assay and real-time PCR were comparable for the detection of FLp53 and Δ40p53 mRNA in FF tissue, a linear regression analysis was performed on the relative expression levels of these genes, derived from the two different assays. The relative expression level of FLp53 was significantly correlated between the two methods (R^2^ = 0.9927, p = 0.0031; Fig 2A) in FF tissues, however, no significant correlation was observed for Δ40p53 expression (R^2^ = 0.4429, p = 0.3345; Fig 2B). Notably, the expression of Δ40p53 in the QuantiGene 2.0 Assay was not above the limit of detection in FF tissues (Table 2).

For the detection of FLp53 mRNA expression, a positive correlation was observed when comparing the relative expression of FLp53 in FFPE to FF samples using real-time PCR (Fig 2C; R^2^ = 0.8753, p = 0.0644). However, as shown in Fig 2D, there was no correlation with FLp53 expression between FF and FFPE using the QuantiGene 2.0 Assay (R^2^ = 0.0002, p = 0.9863). The QuantiGene 2.0 Assay lacks the sensitivity to detect levels of FLp53 mRNA above the limit of detection (Table 2) in FFPE tissues, and this may have contributed to the lack of correlation between the FFPE and FF samples using the hybridization method.

We next compared the relative expression of FLp53 in FFPE between the two different methods. There was no correlation between the different assays when examining FLp53 expression in FFPE-derived mRNA (R^2^ = 0.0084, p = 0.9081) (Fig 2E), and this supports our findings that the QuantiGene 2.0 Assay was unable to accurately measure FLp53 mRNA expression in FFPE tissues. Therefore, the expression of FLp53 using real-time PCR was highly correlated with the QuantiGene 2.0 Assay in the FF samples, however the methods were not comparable for the analysis of FFPE samples.

## Discussion

Traditionally, real-time PCR is used to measure gene expression from FF tissue samples. However, the length of the amplicon required, and problems that arise during the production of cDNA, limit the use of this technology for quantitating gene expression from FFPE tissue [12]. In this study, the QuantiGene 2.0 Assay was compared to real-time PCR in the measurement of mRNA expression in a small number of FFPE and matched FF breast cancer samples. Our results showed that the QuantiGene 2.0 Assay was not comparable to real-time PCR when using mRNA from FFPE samples, compared to matched FF tissue. This suggests that the QuantiGene 2.0 Assay was not sensitive enough: 1) to detect the low expressed signal of Δ40p53 in these tissues, either from FF or FFPE-derived material and 2) to detect FLp53 in highly degraded FFPE samples. This suggests that the traditional method of real-time PCR is the most appropriate for the detection of the low expressed Δ40p53 mRNA transcript in FF tissues, and for the detection of FLp53 in tissues that are highly degraded [10].

The lack of sensitivity observed with the QuantiGene 2.0 Assay may be due to the large region covered by the Z-probes. This assay uses a Z-probe structure which binds to smaller 20bp regions consecutively to encompass a larger targeted region. If the RNA is degraded within the areas covered by the Z-probes, they fail to bind consecutively and hence the branched DNA capture Probe Sets will not be able to bind to the Z-probes and amplify the signal. Therefore, the region covered by the Z-probes may be too long for the adequate detection of degraded RNA from FFPE samples. Furthermore, the minimal signal detected for Δ40p53 in both FF and FFPE samples by this assay, at limits below the limit of detection, suggests that the QuantiGene 2.0 Assay lacks the sensitivity required to adequately quantitate the low abundant Δ40p53 isoform. However, these assays were only performed in a small number of tissue samples, and they would need to be repeated in a larger cohort to validate these findings.

A number of studies have quantified the relative mRNA expression of different genes from FFPE tissues using the QuantiGene 2.0 Assay [6, 13]. They also demonstrated high sensitivity using the QuantiGene 2.0 Assay, when compared the real-time PCR, nested RT-PCR and FISH, and have shown its usefulness as an appropriate method for validating gene expression levels from FFPE tissues [6, 14, 15]. However, the limitations surrounding the design of the probes for the detection of low abundance transcripts, such as Δ40p53, do not allow for other alternative probes which may be more sensitive and provide greater signal detection above the limit of detection. Therefore, this study is limited by the fact that the Δ40p53 mRNA sequence is highly homologous to FLp53, and testing other methods of quantification are required to determine the usefulness of measuring Δ40p53 in FFPE tissues. Such methods include digital PCR or RNA in-situ hybridization, but the use of any technology will require extensive optimization to determine their suitability for quantification of lowly expressed, and hard to target, transcripts such as Δ40p53 in FFPE tissues.

## Conclusions

Taken together, this preliminary study has demonstrated that the QuantiGene 2.0 Assay is appropriate for quantitating FLp53 mRNA expression from FF tissues but that it is not sensitive enough for detection of FLp53 from degraded FFPE tissues. Real-time PCR was shown to be the most appropriate method for the quantification of FLp53 from FFPE tissue samples, but neither real-time PCR or the QuantiGene 2.0 Assay were suitable for the quantification of the low abundant Δ40p53 mRNA transcript in FFPE tissues. Other methods need to be investigated to accurately quantitate the relative expression of Δ40p53 mRNA in FFPE tissue samples.

## Supporting Information

S1 Fig Raw data output of the QuantiGene 2.0 Assay. The luminescent signal detected for each sample for the detection of mRNA expression of β-Actin, Δ40p53 and FLp53 in 4 FF or FFPE breast tumour tissues using the FLUOstar Optima plate reader. (A) The plate layout, and detail for each sample is shown below. (B) The relative luminescent unit (RLU) detected for each well. (C) The RLU minus the average assay background (AB) for each target probe set. An assay background was performed in triplicate for each target probe set, which measures the background luminescent signal for each assay in the absence of sample input. Each sample was performed in triplicate for each assay.
(TIF)

S2 Fig Graphical representation of real-time PCR data for the detection of housekeeping genes in FF and FFPE breast tumour samples. Gene expression analysis of 4 FF and matched FFPE breast tumour samples in the detection of β-Actin (FF) and β2M (β2-Microglobulin; FFPE). Data is shown as the log (ΔRn) against the PCR cycle number. Each sample was analysed in triplicate.
(TIF)

S3 Fig Graphical representation of real-time PCR data for the detection of Δ40p53 expression in FF and FFPE breast tumour samples. Gene expression analysis of 4 FF and matched FFPE breast tumour samples in the detection of Δ40p53. Data is shown as the log (ΔRn) against the PCR cycle number. Each sample was analysed in triplicate.
(TIF)

S4 Fig Graphical representation of real-time PCR data for the detection of FLp53 expression in FF and FFPE breast tumour samples. Gene expression analysis of 4 FF and matched FFPE breast tumour samples in the detection of FLp53. Data is shown as the log (ΔRn) against the PCR cycle number. Each sample was analysed in triplicate.
(TIF)
