# Supplementary figures and images for: Comparison of the QuantiGene 2.0 Assay and Real-Time RT-PCR in the Detection of p53 Isoform mRNA Expression in Formalin-Fixed Paraffin-Embedded Tissues- A Preliminary Study
Source: PLoS One. 2016 Nov 10;11(11):e0165930. doi: 10.1371/journal.pone.0165930 (PMC5104334; doi:10.1371/journal.pone.0165930)

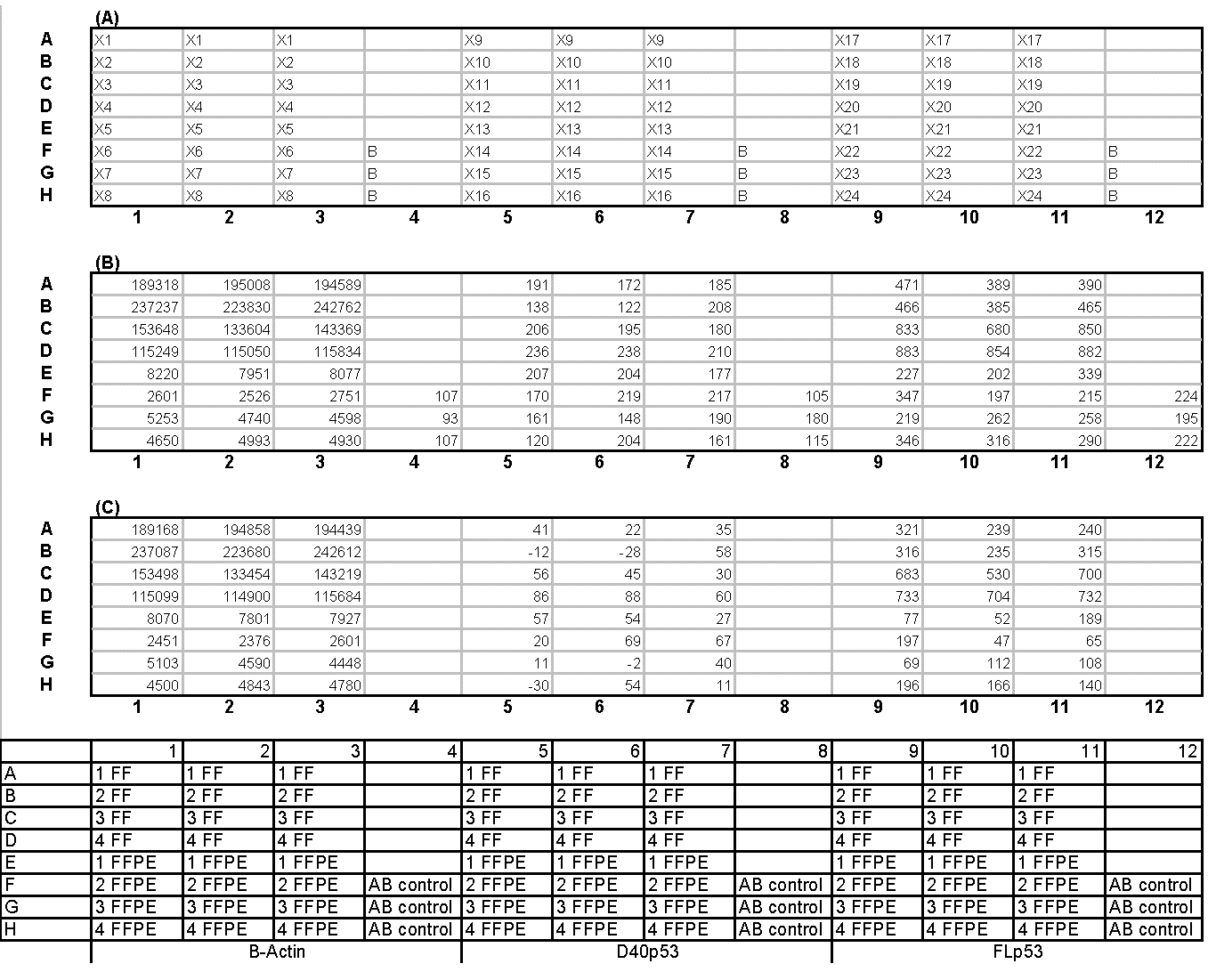

Supplement: S1 Fig — The luminescent signal detected for each sample for the detection of mRNA expression of β-Actin, Δ40p53 and FLp53 in 4 FF or FFPE breast tumour tissues using the FLUOstar Optima plate reader. (A) The plate layout, and detail for each sample is shown below. (B) The relative luminescent unit (RLU) detected for each well. (C) The RLU minus the average assay background (AB) for each target probe set. An assay background was performed in triplicate for each target probe set, which measures the background luminescent signal for each assay in the absence of sample input. Each sample was performed in triplicate for each assay. (TIF) [file pone.0165930.s001.tif]

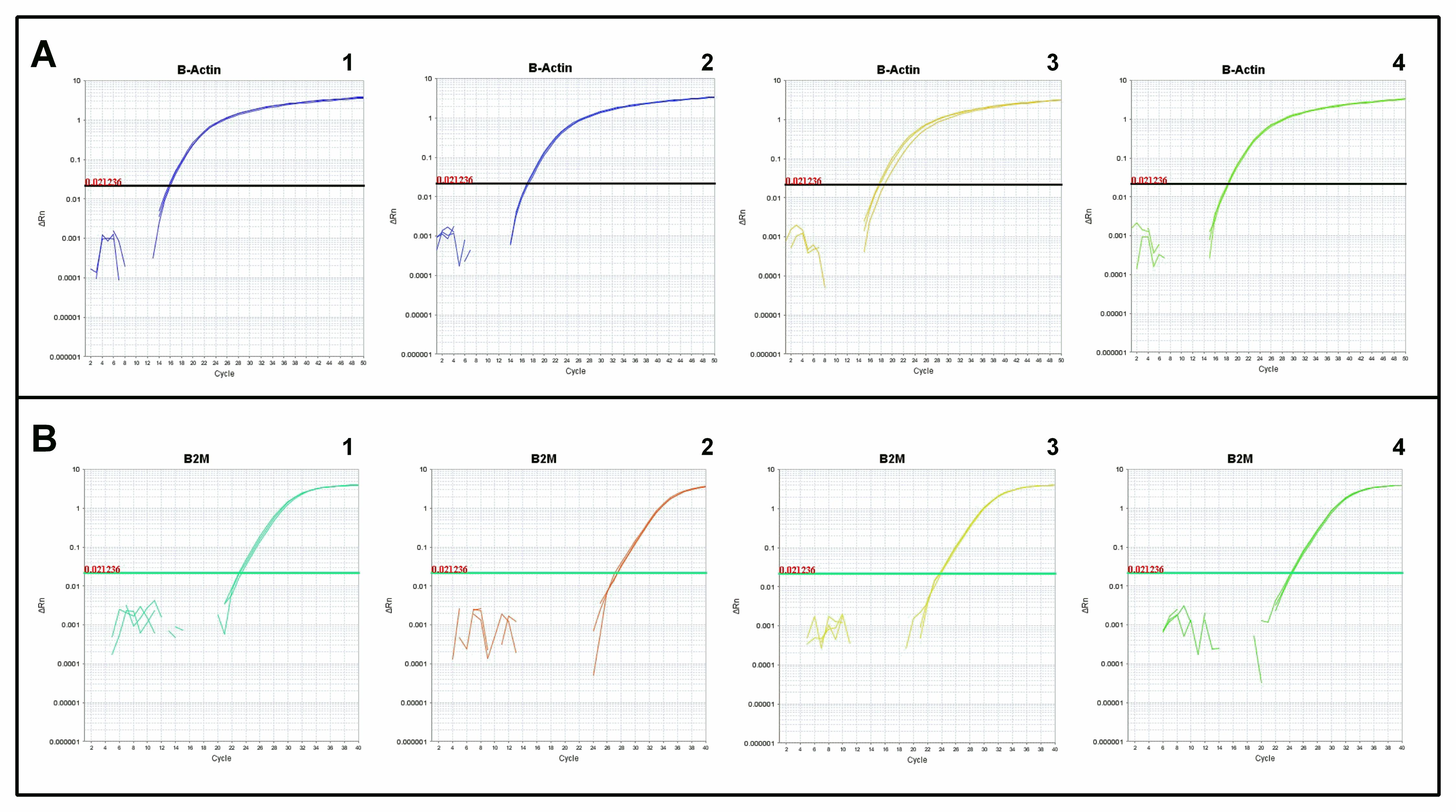

Supplement: S2 Fig — Gene expression analysis of 4 FF and matched FFPE breast tumour samples in the detection of β-Actin (FF) and β2M (β2-Microglobulin; FFPE). Data is shown as the log (ΔRn) against the PCR cycle number. Each sample was analysed in triplicate. (TIF) [file pone.0165930.s002.tif]

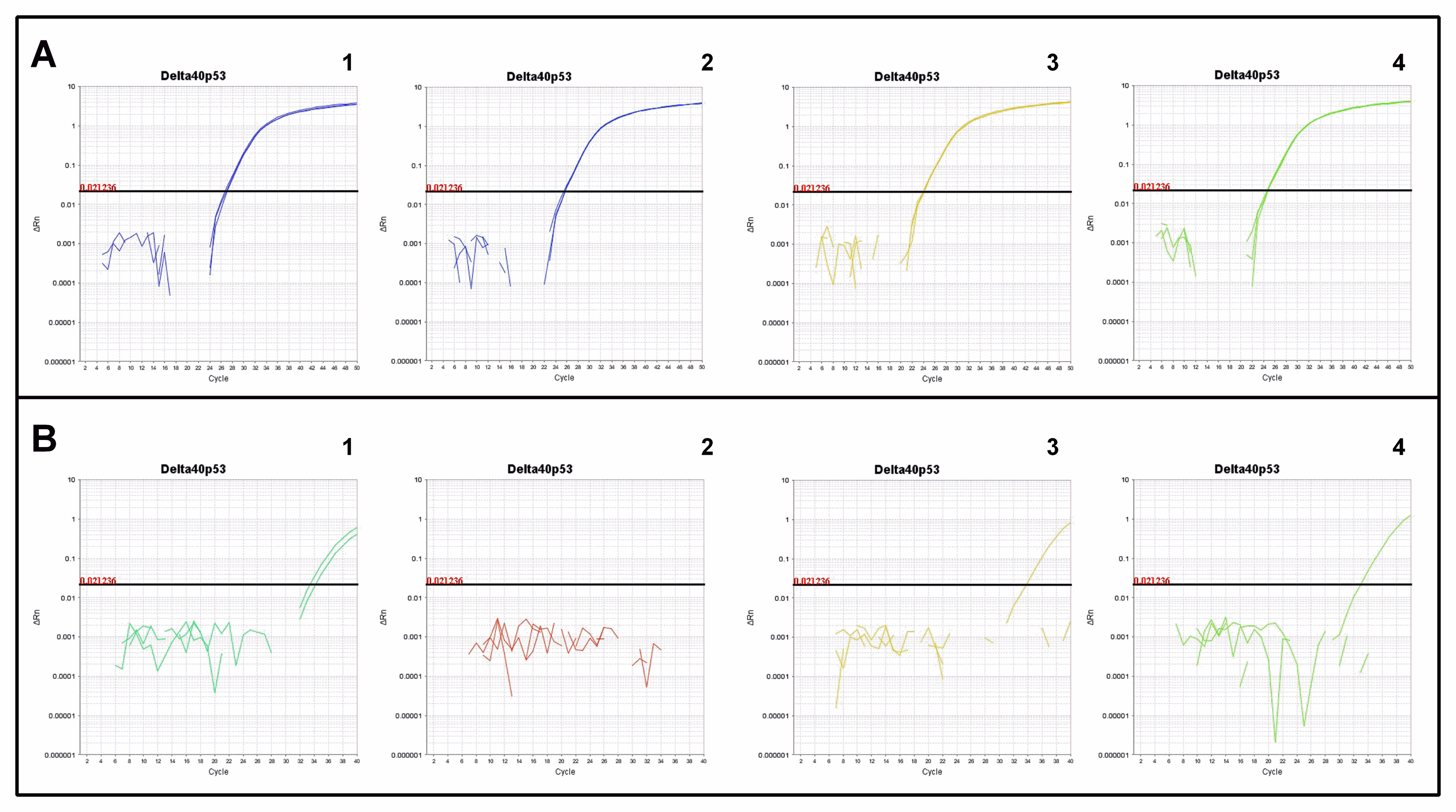

Supplement: S3 Fig — Gene expression analysis of 4 FF and matched FFPE breast tumour samples in the detection of Δ40p53. Data is shown as the log (ΔRn) against the PCR cycle number. Each sample was analysed in triplicate. (TIF) [file pone.0165930.s003.tif]

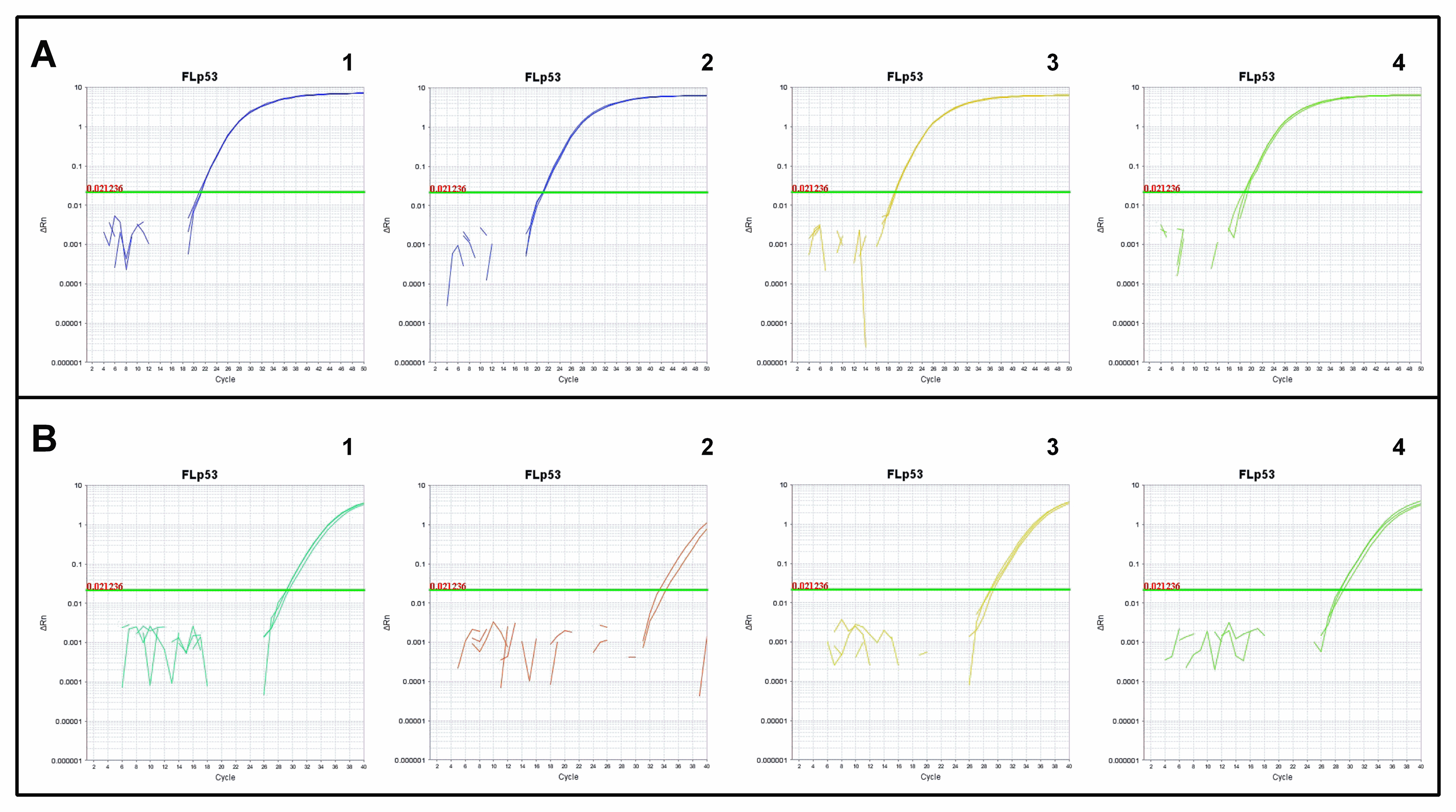

Supplement: S4 Fig — Gene expression analysis of 4 FF and matched FFPE breast tumour samples in the detection of FLp53. Data is shown as the log (ΔRn) against the PCR cycle number. Each sample was analysed in triplicate. (TIF) [file pone.0165930.s004.tif]
